# Clozapine-Related Thromboembolic Events

**DOI:** 10.7759/cureus.16883

**Published:** 2021-08-04

**Authors:** Elisa Pallares Vela, Prashil Dave, Ivan Cancarevic

**Affiliations:** 1 General Medicine, California Institute of Behavioral Neurosciences & Psychology, Fairfield, USA; 2 General Practice, California Institute of Behavioral Neurosciences & Psychology, Fairfield, USA; 3 Internal Medicine, California Institute of Behavioral Neurosciences & Psychology, Fairfield, USA

**Keywords:** clozapine, antipsychotics, atypical antipsychotics, pulmonary embolism, venous thrombosis, thrombosis, thrombotic events

## Abstract

Clozapine is an atypical antipsychotic used for treatment-resistant schizophrenia. Venous thromboembolism (VTE) is a rare side effect of clozapine which can be fatal. This article summarizes current evidence regarding the risk of VTE associated with the use of clozapine. We performed a PubMed (MeSH) and Google Scholar search for the last two decades. Studies or case reports performed in humans were included in the review, of which 42 case reports of patients taking clozapine at VTE onset were included in the analysis of this review. According to the articles reviewed, the mean age was 42.9 years, with more males (71.43%) than females (28.57%). The average clozapine dose was 285.62 mg/day. VTE onset occurred within the first six months in 71.8% of the cases. Overall, 70.37% of the patients had comorbidities, and 87.5% had risk factors for VTE. In total, 68.57% were prescribed other medications at VTE onset, and 60% were being treated with another antipsychotic concomitantly. Finally, 32.5% of the patients died, while 67.5% survived. In 60% of the cases, clozapine was discontinued after VTE. In our literature review, we observed that among clozapine users, VTE occurred at a wide dose range, and most of the events occurred within the first six months. As many patients who are prescribed clozapine have risk factors for VTE, the risk should be considered at the time of prescribing. Further research should be conducted to elucidate the risk of VTE in clozapine users and the benefits of thromboprophylaxis.

## Introduction and background

Schizophrenia is a mental disorder that affects approximately 20 million people worldwide [1]. Despite its low prevalence, schizophrenia is a severe mental illness that may profoundly impact the individual [1]. It is a disease with complex genetics and not well-elucidated pathophysiology where environmental factors may play an important role [2]. Studies are constantly being conducted to further understand this mental disorder.

Pharmacological and nonpharmacological therapies are available and can be beneficial. Antipsychotics, both typical and atypical, have been widely used for the treatment of this disorder [2]. Atypical antipsychotics have been found to have a better safety profile when talking about neurological side effects than typical antipsychotics; nevertheless, no significant differences have been observed regarding the effectiveness of the treatment of positive symptoms [2]. The choice of long-term therapy depends upon the prior history of response and the avoidance of side effects [2].

For patients who have refractory schizophrenia, clozapine, an atypical antipsychotic, is the preferred treatment pathway [2,3] and has shown better response rates than other drugs [2,3]. Clozapine is a dibenzodiazepine that interacts with different subtypes of dopaminergic, serotoninergic, adrenergic, histaminergic, and muscarinic receptors [4]. Apart from presenting fewer neurological side effects, it has been observed to reduce mortality rates for suicide in patients with schizophrenia [4]. In contrast, there are many adverse effects, with one of the rare ones being pulmonary embolism (PE) [4]. PE contributes to increased mortality in patients prescribed clozapine [4].

Venous thromboembolism (VTE) is a multifactorial disease that encircles PE and deep vein thrombosis (DVT) and is one of the three leading causes of death from cardiovascular diseases [5,6]. It is estimated that there are 100,000 to 180,000 deaths annually in the United States due to PE, and it is the most common preventable cause of death among hospitalized patients [7]. Well-described risk factors for VTE include major surgery (general or orthopedic), lower extremity paralysis, lower extremity fractures, multiple trauma, cancer, prior VTE, age (>40 years), obesity, immobility, oral contraceptives or estrogen treatment, pregnancy or postpartum, genetic blood conditions, sedentarism, and family history to VTE, among others [8].

Thrombotic events have been observed in patients taking clozapine [3,4]. However, there is insufficient evidence to hold clozapine accountable for thrombotic events or to know if VTE develops as a consequence of weight gain and sedation, and if it is positively correlated with PE [3,4].

Despite existing research, it remains unclear whether clozapine treatment causes thrombotic events to occur or if the disease itself puts these patients at a higher risk of developing thrombotic events. The mechanisms underlying the development of thrombotic events in schizophrenic patients and those taking clozapine are not yet elucidated. This study aims to perform a literature review of the latest evidence and case reports intending to unearth the relationship between clozapine and thrombotic events.

Methodology

We performed a comprehensive PubMed (MeSH) and Google Scholar search for the last two decades. We used the keywords “clozapine,” “antipsychotics,” or “atypical antipsychotics” and combined them using the Boolean operator “and” with the words “pulmonary embolism,” “venous thrombosis,” “vein thrombosis,” “thrombosis,” or “thrombotic events.” We included human-only studies or case reports with an available abstract or a full-text article in English, Spanish, or French languages. Only case reports of patients who were taking clozapine at the onset of VTE were included. We excluded articles or case reports that did not meet these criteria. Overall, we found 42 case reports published in the last two decades.

## Review

VTE is a rare adverse event associated with clozapine use [4,9]. There is consistent evidence with regards to a higher risk of VTE among patients prescribed clozapine, namely, between three-fold and 27.5-fold risk of VTE compared to the general population [6,10]. According to Sackey et al., PE is the second leading cause of death after external factors such as accidents or suicide [11]. A study of the World Health Organization database from 79 countries on adverse drug reactions showed 754 cases of VTE during antipsychotic treatment, out of which approximately 375 were related to clozapine use [12]. This review included a total of 42 case reports published in the last two decades (Table 1) [10-13,26-49]. We included cases of clozapine users who developed VTE including PE and DVT.

**Table 1:** Clozapine-related VTE BMI: body mass index; BPH: benign prostatic hyperplasia; DM: diabetes mellitus; HT: hypertension; NA: not available; PE: pulmonary embolism; VTE: venous thromboembolism

Reference number	Publication year	Author	Age	Sex	Dose (mg/day)	Treatment duration	Outcome	Risk factors for VTE	Clozapine discontinuation	Comorbidities	Additional medication at VTE onset	Another antipsychotic at VTE onset
[10]	2018	Tseng and Huang	41	Male	175	24 months	Survived	Yes	No	Obese (BMI 39 kg/m^2^)	Lithium	No
[11]	2017	Sackey et al.	60	Male	500	3 days	Survived	Yes	No	Transient ischemic attacks, HT, hypothyroidism	No	No
[12]	2000	Hägg et al.	59	Male	300	14 days	Death	Yes	-	NA	Phenoxymethylpenicillin	No
[12]	2000	Hägg et al.	26	Male	500	20 months	Death	NA	-	NA	Cyproterone acetate	No
[12]	2000	Hägg et al.	38	Female	150	14 days	Death	NA	-	NA	Haloperidol	Yes
[12]	2000	Hägg et al.	53	Male	100	NA	Death	Yes	-	NA	Clomipramine	No
[12]	2000	Hägg et al.	33	Male	200	3 months	Death	NA	-	NA	Lactulose, propantheline	No
[12]	2000	Hägg et al.	36	Male	200	3 months	Survived	NA	NA	NA	Erythromycin	No
[12]	2000	Hägg et al.	54	Male	500	15 days	Survived	Yes	NA	NA	Perphenazine, levomepromazine, amitriptyline	Yes
[12]	2000	Hägg et al.	29	Male	400	21 days	Survived	NA	NA	NA	Clonazepam, carbamazepine	No
[12]	2000	Hägg et al.	30	Male	200	3 months	NA	NA	NA	NA	Carbamazepine, orphenadrine, thioridazine	Yes
[12]	2000	Hägg et al.	25	Female	400	NA	Survived	Yes	NA	NA	Levonorgestrel/ethinyloestrdiol, diazepam, terbutaline, budesonide	No
[12]	2000	Hägg et al.	42	Male	75	24 months	Survived	Yes	NA	NA	Biperiden, flupentixol, lorazepam, clomipramine	Yes
[13]	2016	Gami et al.	40	Male	350	6.42 months	Death	Yes	-	Chronic knee pain	Omeprazole, ibuprophen	No
[26]	2000	Coodin and Ballegeer	30	Male	150	12 days	Survived	NA	Yes	NA	NA	NA
[27]	2000	Suttmann et al.	33	Female	250	12 days	Survived	Yes	Yes	Factor V Leiden mutation	NA	NA
[28]	2000	Maynes	30	Male	400	5 months	Survived	NA	Yes	NA	NA	NA
[29]	2001	Ihde-Scholl et al.	29	Male	300	1.4 months	Death	NA	-	NA	NA	NA
[30]	2003	Pan et al.	58	Male	250	5 days	Survived	Yes	Yes	PE 2 years prior	No	No
[31]	2003	Selten and Büller	28	Male	400	10 days	Survived	NA	No	NA	NA	NA
[32]	2003	Yang et al	31	Male	100	2 months	Death	No	-	No	No	No
[33]	2004	Farah et al.	47	Female	300	26 months	Death	Yes	-	Anemia	Paroxetine	Yes
[34]	2006	O’Luanaigh et al.	52	Male	400	9.6 months	Survived	Yes	Yes	No	No	No
[35]	2008	Srihari et al.	45	Male	NA	6 months	Survived	Yes	No	Heavy smoker	No	No
[36]	2008	Vayá et al.	56	Male	125	2 months	Survived	Yes	NA	Prothrombin G20210A mutation carrier	NA	NA
[37]	2009	Yeh and Lee	51	Female	300	15 days	Survived	Yes	Yes	Alcohol abuse, HT, hyperlipidemia, heavy smoker, thyroiditis	Nicotine gum, lithium, olanzapine, valproic acid, octreotide, atropine/diphenoxylate, cholestyramine, tolterodine, levothyroxine, metoprolol tartrate	Yes
[38]	2009	Ul-Haq and Holland	41	Female	450	5 months	Death	Yes	-	No	Lithium, zopiclone, escitalopram, chlorpromazine, and risperidone	Yes
[38]	2009	Ul-Haq and Holland	64	Female	200	21 days	Survived	Yes	Yes	No	NA	NA
[38]	2009	Ul-Haq and Holland	50	Male	500	4 months	Survived	Yes	Yes	No	Amisulpride, mirtazapine, procyclidine	Yes
[39]	2011	Joksovic and Chiles	52	Male	300	17 days	Survived	Yes	Yes	Mild obesity, hyperlipidemia, HT, and BPH	Divalproex sodium, perphenazine, risperidone, simvastatin, furosemide, finasteride, tamsulosin	Yes
[40]	2011	Tripp	58	Female	175	1.4 months	Survived	Yes	NA	Pneumonia one week prior	Quetiapine, moxifoxacin, ibuprophen, acetaminophen, alendronate	Yes
[40]	2011	Tripp	30	Male	700	3 months	Survived	Yes	No	Sickle cell disease, VTE at 12 years old, smoker	No	No
[40]	2011	Tripp	63	Female	200	22 days	Survived	Yes	No	Smoker, HT, asthma, osteoarthritis, overweight (BMI 29.1)	Aspirin, haloperidol, benztropine, famotidine, clonidine	Yes
[41]	2012	Suljemanpasic and Fisekovic	56	Female	400	NA	NA	Yes	NA	Obesity, HT, hyperlipidemia	Chlorpromazine, haloperidol, haloperidol decanoate	Yes
[42]	2013	Chate et al.	39	Male	150	8.6 months	Survived	No	Yes	No	No	No
[43]	2013	Munoli et al.	34	Male	50	84 months	Survived	No	Yes	No	No	No
[44]	2013	Hu et al.	22	Male	500	24 months	Death	No	-	No	No	No
[45]	2014	Schmidinger and Hofer	63	Male	50	14 days	Survived	Yes	Yes	Alcohol and benzodiazepines dependence, hepatic steatosis, overweight (BMI 29.03)	Lorazepam, gabapentin, levofloxacin	No
[46]	2015	Marinkovic and Rancic	26	Female	NA	13.2 months	Death	Yes	-	Postpartum	Haloperidol, lamotrigin, diazepam, temazepam	Yes
[47]	2016	Goh and John	31	Female	300	18 days	Survived	Yes	No	Obese, smoker, poor mobility	No	No
[48]	2018	Li et al.	62	Male	75	1 month	Death	Yes	-	Type 2 DM, acid reflux, tobacco, and cannabis use every day	No	No
[49]	2020	Waters et al.	56	Male	350	20 days	Survived	Yes	No	HT, BPH, type 2 DM, obese (BMI 30 kg/m^2^)	Significant polypharmacy	Yes

Age

In our review, the mean age was 42.92 years (SD = 13) (Table 1). In 23 (54.76%) case reports, patients were 40 years old or more. According to Gami et al., antipsychotic agents are associated with VTE in the elderly, and Letmaier et al. reported that patients aged 65 and older with mood disorders have higher VTE incidence and concluded that clinicians should consider antipsychotic exposure as a risk factor for VTE in this population [13,14]. In contrast, Keijer et al. conducted a study including 111,818 patients aged 60 or older who were on antipsychotic treatment and found no evidence suggesting higher VTE risk than nonusers [15]. According to the American Heart Association (AHA), being 40 years old raises the probability of a VTE, and this risk doubles with each decade [8], independent of antipsychotic use.

Sex

In our review, there were 12 (28.57%) females and 30 (71.43%) males, showing an increased incidence in male antipsychotic users rather than female users (Table 1). These findings are similar to those of previous reviews which found between 63.3% and 75% of patients were males [9,12,16]. In a meta-analysis that included a total of 31,514,226 subjects from 22 studies, Dai et al. observed patients taking first- and second-generation antipsychotics [17]. These patients were at a higher risk of VTE than those who were not prescribed antipsychotics, and females taking antipsychotics had a higher risk of PE than males [17]. In the same study, the authors reported that clozapine users have significantly increased VTE risk [17].

Comorbidities and risk factors for venous thromboembolism

Out of the 42 case reports, 15 did not report patient comorbidities (Table 1). Out of the remaining 27 reports that did, 19 (70.37%) patients had one or more comorbidities and eight (29.63%) patients did not have any comorbidities. Out of the 19 patients who had comorbidities, 10 (52.63%) had comorbidities that are described as risk factors for VTE by the AHA (mutations associated with blood clotting, obesity, previous thrombotic events, postpartum) (Table 2) [8]. When describing risk factors for VTE, including age, we could not find information in 10 case reports; of the remaining 32 reports, only four (12.5%) had no reported risk factors for VTE, while 28 (87.5%) reported risk factors.

**Table 2:** Risk factors for VTE. VTE: venous thromboembolism

Risk factor	Observation
Major surgery (general or orthopedic)	
Low extremity paralysis	
Fracture of long bones, hip, or pelvis	
Multiple trauma	
Cancer	Chemotherapy and surgery further increase the risk
Prior VTE	
Age	>40-year-olds are at a higher risk. Risk doubles with each decade
Obesity	Two times the risk as people with normal weight. The higher the weight, the higher the risk
Immobility	
Oral contraceptives or estrogen therapy	
Family history of VTE	Especially in first-degree relatives
Sedentarism	
Genetic blood conditions that affect clotting	
Pregnancy and postpartum	Twin gestation, obesity, older maternal age, and concomitant illness elevate the risk

According to Ronaldson, patients with mental illnesses usually have risk factors for VTE; however, it is arguable whether VTE is attributable to the pharmacological properties of clozapine or the risk factors of clozapine recipients [18]. Till and Silva reported a case where clozapine was successfully administered in a patient with a previous myocardial infarction and PE [19]. After 94 days of initiating clozapine, the patient’s seclusion was terminated after 1,046 days [19]. The most appropriate attitude toward a psychiatric patient treated with clozapine is not to withdraw the medication and switch to another option but to manage the risk factors predisposing to cardiovascular disease and work as a multidisciplinary group [18,19].

Dose

In our review, two case reports did not report dosage (Table 1). Of the remaining 40, the mean dose was 285.62 mg, with 50 mg being the lowest and 700 mg the highest dose, similar to the findings of Poudyal and Lohani [9]. According to Subramanian et al., doses of 300 mg or less per day are considered low, and doses >300 mg per day are considered high [20]. Out of these 40 patients, 19 (47.5%) had a low dose (>300 mg) and 21 (52.5%) had a high dose (300 mg or more), with a similar distribution. Some studies concluded that most antipsychotic-related VTE appears to be dose-dependent, but no specific dose threshold has been established for clozapine users [11,21]. In contrast to these findings, Kleijer et al. did not find any association between dosage and VTE, and Sarvaiya et al. showed that VTE related to clozapine treatment appears to be dose-independent [15,16].

Time of onset

In our review, three case reports did not specify when VTE occurred regarding the time of clozapine treatment. Out of the other 39 cases, 28 (71.8%) occurred within the first six months of treatment, and the remaining 11 (28.2%) occurred after six months of treatment (Table 1). The mean time of onset was 7.49 months, with the earliest event reported on day three and the latest at 84 months of use. These findings are consistent with those of Sarvaiya et al. [16]. In the case report by Sackey et al., the patient had a prescription of 125 mg, which was well tolerated, but he took five 100 mg tablets instead of five 25 mg tablets for three days before the event occurred [11]. It may suggest that higher doses have a higher risk of developing VTE.

According to Ronaldson, therapy onset with an antipsychotic in the previous 24 months was associated with a 32% increased risk for VTE in antipsychotic users, and in the first three months of therapy, the risk was doubled compared to non-users [18]. She stated that among those initiated on clozapine, 16 per 1,000 developed VTE, and those initiated on other antipsychotics developed VTE in 20 per 1,000 patients [18]. Wilkowska et al. reported that the approximate incidence of VTE among antipsychotic users is 4/1,000 in all age groups and 10/10,000 in patients older than 65 years after the first year of treatment [22]. Jönsson et al. observed that the risk is especially relevant in the first three months of treatment onset, suggesting that the medication itself is a more significant risk factor than the underlying disease [6].

Other medication and concomitant antipsychotic use

Out of the 42 cases, seven did not report on other medications (Table 1). Of the remaining 35 cases, 24 (68.57%) patients took other medications, while 11 (31.43%) did not. Among these 35 patients, 17 (48.57%) had polypharmacy, taking three or more medications at VTE onset, and 21 (60%) were taking another antipsychotic concomitantly at VTE onset. First and second-generation antipsychotics have been related to VTE [6,13,18]. It is difficult to ascertain which antipsychotic has the most substantial relationship with a thrombotic event in a patient taking more than one antipsychotic that has been related to VTE.

Outcome and discontinuation of clozapine

Out of the 42 case reports, two did not specify the patient’s outcome (Table 1). Of the remaining 40 cases, 27 (67.5%) patients survived, while 13 (32.5%) died. Studies have reported mortality rates of 26.21-41.66% [9,12,16]. Sarvaiya et al. reported that clozapine doses were similar in the group who died and the group who survived [16]. Of the 27 patients reported to survive in our case series, seven did not specify if the treatment was continued or discontinued. Of the 20 reports for which we had the information, we observed that clozapine was discontinued in 12 (60%) cases and continued in eight (40%). Interestingly, we can observe that in the last five years, the therapeutic behavior has changed as most of the earlier cases had it discontinued and the recent ones have continued with the treatment (Table 1).

Some authors recommend stopping the treatment if a VTE is suspected in a clozapine user [6,12]. Others have suggested prophylactic antithrombotic treatment for patients who have other risk factors for developing VTE [6]. The use of prophylaxis is controversial; in a study, Therasse et al. concluded that DVT incidence was not low despite prophylaxis in psychiatric patients [23]. Another study found that not using anticoagulants was not associated with an increased risk of VTE [24]. Verdoux et al. suggested that discontinuation may be justified in case of VTE recurrence [25].

Managing individual patient risk factors can modify VTE risk in clozapine takers, and continuation versus discontinuation after a PE event should be decided after considering each patient’s risk factors, benefits, and disadvantages of continuation [13,16]. We found similar opinions in studies by Ronaldson and Till and Silva, who suggested multidisciplinary teamwork [18,19].

Limitations

Findings from our study depend on the methodological quality of each case report. Furthermore, our outcomes may be limited by missing or incomplete data. There were limited data on patients who continued clozapine use after having a VTE regarding new events. We only included English, Spanish, and French articles for our search; therefore, other case reports may not be included in our study. The lack of a systematic research strategy is also a potential limitation.

## Conclusions

Clozapine has proven to be highly effective in the treatment of resistant schizophrenia. VTE occurs at a wide dose range and more extensive studies should be performed to assess if it is a dose-dependent or independent event. VTE onset appears to be higher during the first six months of treatment. A considerable number of patients prescribed clozapine have comorbidities and at least one risk factor for VTE. Many patients on clozapine also take other medications, including other antipsychotics, that have been related to VTE. Discontinuation of clozapine after VTE appears to have diminished in the last years. It is essential to consider the risks and benefits of clozapine and work as a multidisciplinary group before making decisions, considering that, often, clozapine is the best option for a patient to have a better quality of life. Knowing that VTE is a potentially lethal event, it would be recommended to direct more extensive prospective cohort studies to elucidate the actual relationship so that multidisciplinary teams can write guidelines regarding decision-making on clozapine discontinuation and thromboprophylaxis.
